# Natural Language Processing–Enabled and Conventional Data Capture Methods for Input to Electronic Health Records: A Comparative Usability Study

**DOI:** 10.2196/medinform.5544

**Published:** 2016-10-28

**Authors:** David R Kaufman, Barbara Sheehan, Peter Stetson, Ashish R Bhatt, Adele I Field, Chirag Patel, James Mark Maisel

**Affiliations:** ^1^ Department of Biomedical Informatics Arizona State University Scottsdale, AZ United States; ^2^ Health Strategy and Solutions Intel Corp Santa Clara, CA United States; ^3^ Internal Medicine Memorial Sloan Kettering Cancer Center New York, NY United States; ^4^ ZyDoc Medical Transcription LLC Islandia, NY United States; ^5^ Department of Neurology & Neurological Sciences Stanford School of Medicine Stanford University Palo Alto, CA United States

**Keywords:** electronic health records, natural language processing, medical transcription, user-computer interface

## Abstract

**Background:**

The process of documentation in electronic health records (EHRs) is known to be time consuming, inefficient, and cumbersome. The use of dictation coupled with manual transcription has become an increasingly common practice. In recent years, natural language processing (NLP)–enabled data capture has become a viable alternative for data entry. It enables the clinician to maintain control of the process and potentially reduce the documentation burden. The question remains how this NLP-enabled workflow will impact EHR usability and whether it can meet the structured data and other EHR requirements while enhancing the user’s experience.

**Objective:**

The objective of this study is evaluate the comparative effectiveness of an NLP-enabled data capture method using dictation and data extraction from transcribed documents (NLP Entry) in terms of documentation time, documentation quality, and usability versus standard EHR keyboard-and-mouse data entry.

**Methods:**

This formative study investigated the results of using 4 combinations of NLP Entry and Standard Entry methods (“protocols”) of EHR data capture. We compared a novel dictation-based protocol using MediSapien NLP (NLP-NLP) for structured data capture against a standard structured data capture protocol (Standard-Standard) as well as 2 novel hybrid protocols (NLP-Standard and Standard-NLP). The 31 participants included neurologists, cardiologists, and nephrologists. Participants generated 4 consultation or admission notes using 4 documentation protocols. We recorded the time on task, documentation quality (using the Physician Documentation Quality Instrument, PDQI-9), and usability of the documentation processes.

**Results:**

A total of 118 notes were documented across the 3 subject areas. The NLP-NLP protocol required a median of 5.2 minutes per cardiology note, 7.3 minutes per nephrology note, and 8.5 minutes per neurology note compared with 16.9, 20.7, and 21.2 minutes, respectively, using the Standard-Standard protocol and 13.8, 21.3, and 18.7 minutes using the Standard-NLP protocol (1 of 2 hybrid methods). Using 8 out of 9 characteristics measured by the PDQI-9 instrument, the NLP-NLP protocol received a median quality score sum of 24.5; the Standard-Standard protocol received a median sum of 29; and the Standard-NLP protocol received a median sum of 29.5. The mean total score of the usability measure was 36.7 when the participants used the NLP-NLP protocol compared with 30.3 when they used the Standard-Standard protocol.

**Conclusions:**

In this study, the feasibility of an approach to EHR data capture involving the application of NLP to transcribed dictation was demonstrated. This novel dictation-based approach has the potential to reduce the time required for documentation and improve usability while maintaining documentation quality. Future research will evaluate the NLP-based EHR data capture approach in a clinical setting. It is reasonable to assert that EHRs will increasingly use NLP-enabled data entry tools such as MediSapien NLP because they hold promise for enhancing the documentation process and end-user experience.

## Introduction

### Electronic Health Records and Data Entry

Electronic health records (EHRs) permeate most medical practices in the United States [1]. A promising feature of EHRs is that they provide machine-readable structured data that can be stored electronically, so that patient-centered information can be reviewed, retrieved, reported, and shared in real time to facilitate patient care. Although narrative data entry supports a measure of flexibility, structured data entry confers a number of advantages such as ready access to clinical decision support and interoperability between EHRs and health information exchanges. To achieve the full complement of these benefits, health care providers must generate clinical notes and reports in both human-readable and machine-readable formats. This adds effort to the documentation workflow [2,3] and requires new computer skills of physicians. The additional work required has led to a growing number of data entry alternatives [4], which is the subject of this paper.

Since their inception, EHRs “have been proposed as a means for improving availability, legibility, and completeness of patient information” [5]. The potential benefits of EHRs as instruments of patient care are widely recognized. Spurred by the 2009 US Health Information Technology for Economic and Clinical Health (HITECH) Act and accompanying incentives for providers to use EHRs, advancements in EHR technologies and implementation in the United States have grown rapidly. Approximately 78% of office-based physicians reported using some form of EHR in 2013 [6]. The role of EHRs is now considered integral to achieving federal health care goals, as expressed in the Meaningful Use mandate, for example. This has compelled physicians to adapt to new methods of documentation with concomitant changes to clinical workflow. This has resulted in great uncertainty about the impact of these requirements on the effective application of EHR systems [7].

As EHR implementations continue, physicians frequently express dissatisfaction with EHR documentation methods and usability [8]. Problems associated with usability impact not only the quality of patient records but can even contribute to compromised patient safety [9,10]. EHR documentation places ever-increasing demands on clinicians’ time, which contributes further to diminished quality of documents (eg, replete with irrelevant, redundant, and erroneous information) and physician dissatisfaction. EHR usability is a complex problem involving a multitude of factors [11-13], many of which are beyond the scope of this study. The focus in this paper is on the usability of data capture methods designed to enhance and potentially alleviate some of the burden resulting from manual input methods.

### Natural Language Processing–Based Solutions

Natural language processing (NLP) has emerged as a viable solution for clinical data capture. Many challenges remain for keyboard-and-mouse entry, namely, having to type text and negotiate the often unwieldy EHR interface to record information in structured fields. This is exacerbated by the fact that much of the EHR content continues to be unstructured [3,14]. A 2015 American Medical Informatics Association report identified time-consuming data entry as a problem with EHRs and recommended improving the EHR interface by allowing “multiple modes of data entry to accommodate provider preferences, including voice, typing, clicking, and handwriting recognition” [15].

Although most clinical information in EHRs is stored as unstructured data, such as clinical narrative, its electronic capture or retrieval has been challenging [16]. NLP has the potential to enable the clinician to reduce the documentation burden with the advantages of dictation—efficiency, usability, and quality—and also satisfy the needs for both machine-readable structured data and human-usable rich text in the EHR.

Problems associated with the time required for documentation and usability are well established. However, there is also evidence to suggest that quality of EHR documents (eg, progress reports) is problematic [17,18]. Physician documents often contain redundant, extraneous information or missing and inaccurate patient data [17]. EHR notes are not optimally used to either facilitate clinical communication or enhance patient care [8]. The measure of the quality and completeness of data in the EHR represents a challenging issue [19,20]. Stetson and colleagues [21] developed a tool for quantifying documentation quality, the Physician Documentation Quality Instrument (PDQI), and demonstrated its construct validity and internal consistency reliability. The initial tool consisted of 22 items and was subsequently reduced to a 9-item tool in order to facilitate its real-world application. The instrument can be used to assess the output quality of EHR note modules, as well as explain the components of document areas in need of improvement. Stetson and colleagues [21] assessed the interrater reliability using the intraclass correlation for consistency of average measures on the PDQI-9 total scores and found it to be 0.83 (CI 0.72-0.91). The tool can reliably be used to compare documentation methods and changes in quality resulting from a change in such methods.

In this study, we were focally concerned with testing NLP-enabled dictation-based data capture as a potential solution for relieving the increased burden of documentation. The benchmarks of performance include measures of time, data quality, and usability. According to Cimino [5], “Improvements in the documentation process hold promise for more than simply reduced data entry effort and more readable notes. If impressions and plans can be captured as explicit data elements, using standard terminology, rather than being buried in the narrative text of a note, EHRs could use this information to better support clinical work flow.” As a result of physicians capturing explicit data elements, their clinical reasoning can be made more transparent and more easily available to colleagues caring for the same patient via electronic access to their EHR or data exchange.

### Data Capture Methods

A variety of modalities have been used for creating clinical documentation for EHR data capture or extraction to generate structured, actionable data (ie, data that are consumable, usable, reusable by a computer, and exchangeable with other computer systems in an efficient manner). These modalities include paper-based records transfer; verbal communication; direct entry or direct entry with macros; electronic templates; “Smart Forms”; dictation using speech recognition, sometimes known as voice recognition or continuous speech recognition; transcription or transcription with manual error correction; patient-recorded data (various methods); and hybrids, with or without NLP data capture, also termed “text processing” [22]. Rosenbloom et al observe that in spite of a “profusion of computer-based documentation (CBD) systems that promote real-time structured documentation,” it is a challenge “integrating clinical documentation into workflows that contain EHR systems.” They further note that health care providers prefer the ability to achieve a certain balance by both using a standardized note structure and having the flexibility to use expressive narrative text, facilitated by speech recognition. NLP systems afford that expressivity in developing a patient narrative as well as offering the capability to encode structured notes in a range of clinical document types and forms [22,23].

**Figure 1:**
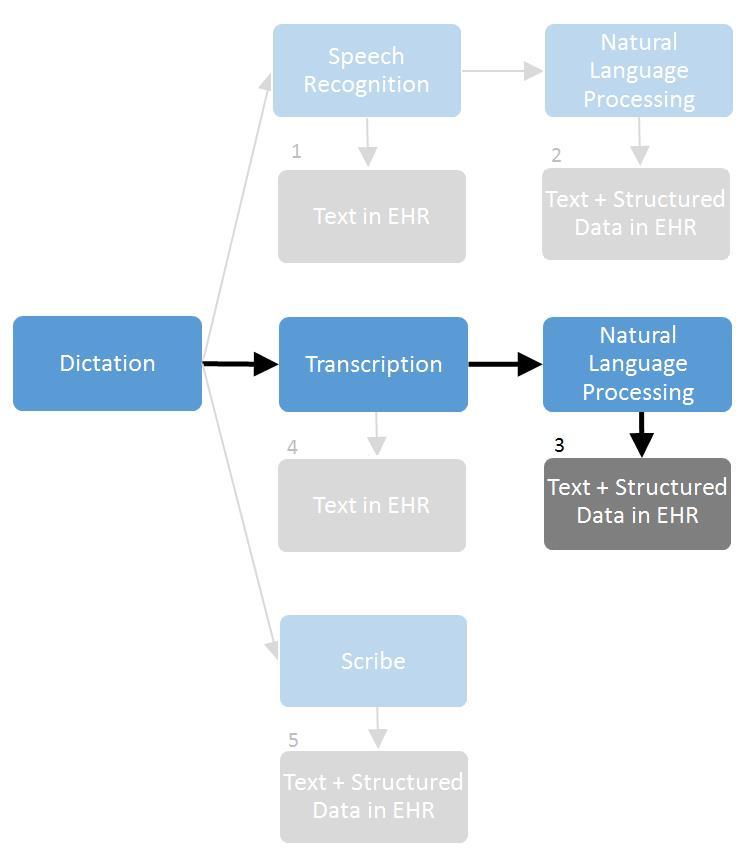
Five dictation-based electronic health record (EHR) data capture methods.

Figure 1 illustrates 5 alternative dictation-based EHR data capture methods. The NLP Entry method used in the study is shown in the center of the figure (labeled as 3), with bold arrows and boxes. Methods 1 and 4 show speech recognition and transcription, respectively, converting dictation to text that is inserted into the EHR. Methods 2 and 3 show NLP being applied to the speech-recognized and transcribed text, respectively, to generate structured data that are inserted into the EHR alongside the text. Method 5 shows a human scribe manually entering text and structured data into the EHR immediately in live time as the physician dictates or at a later time from recorded dictation.

NLP encompasses a family of methods for processing text. These methods have been used for a range of EHR applications [24] including information extraction [25], information retrieval [26], question answering, and text summarization [27]. NLP has also been used for the automatic encoding of narrative text into EHRs [4,28]. NLP and associated technologies used in conjunction with dictation for capturing and structuring medical data have advanced considerably in recent years [4,29].

**Figure 2:**
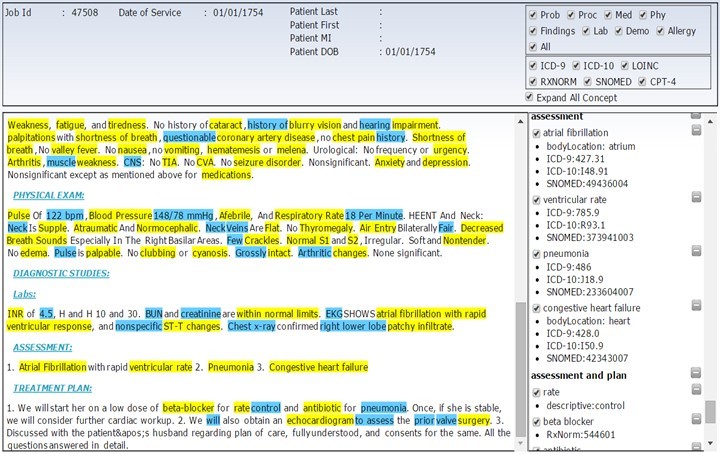
MediSapien NLP application user interface, illustrating the volume of structured data generated by MediSapien NLP. NLP: natural language processing.

Whereas relatively few NLP systems for structured clinical data capture are implemented outside academic medical centers [22], NLP is gaining more traction as a viable commercial technology for populating EHRs. In addition to the MediSapien NLP (ZyDoc Medical Transcription LLC) application used in this study (the user interface for which is shown in Figure 2), there are a limited number of other NLP products being marketed or developed for use with EHR systems to enable NLP Entry (ie, free-text data capture, structuring, and EHR population). For example, both M*Modal [30] and Nuance [31] offer dictation products with voice recognition that structure some data for EHRs. Certain EHR vendors, such as Allscripts, Greenway, and Cerner among others, have integrated the M*Modal or Nuance technologies into their EHRs [32]. Other NLP-based research studies (including one on the interpretation of free-text Papanicolaou test reports for clinical decision support [33] and another on the use of “cognitive analytic tools to gain insight from all types of healthcare information,” including “knowledge-driven decision support” and “data-driven decision support” [34]) demonstrate the increasing importance of NLP in generating and analyzing structured health data.

The NLP engine used by the MediSapien NLP data capture application is the Medical Language Extraction and Encoding System (MedLEE), which was developed at Columbia University in the Department of Biomedical Informatics. MedLEE accepts unstructured clinical text inputs and outputs structured clinical information in a variety of formats [35]. Utilizing clinical lexicons, it is able to normalize clinical concepts in the text to conform to various standard terminologies. It is also able to identify, among other attributes, negation, degrees of certainty, temporal data, and results associated with the identified clinical concepts. MedLEE has been used for a number of data extraction purposes but was not specifically optimized for generating clinical documentation [36]. A commercially available version of MedLEE is now licensed and maintained by Health Fidelity under the product name REVEAL.

Developed by ZyDoc Medical Transcription, the MediSapien NLP data capture application allows doctors to use unstructured dictation to capture structured data in the EHR. MediSapien NLP preprocesses documents, leverages the MedLEE NLP engine, and postprocesses the NLP output using patent-pending processes that augment the NLP engine’s output. It also enables a workflow by which (1) the physician dictates, (2) the dictation is transcribed or subjected to speech recognition, (3) MediSapien NLP generates structured data from the transcription, and (4) the structured data and text are inserted into the EHR, although we simulated the EHR interface in the study.

Figure 2 shows part of a screen from the MediSapien NLP application in which source text is displayed on the left, with medical concepts highlighted, and structured data generated from the source text are displayed on the right. The document included in Figure 2 was selected to illustrate the volume of structured data generated by the MediSapien NLP application; the text was not produced as part of the study presented in this paper. As an indication of the volume of data generated by MediSapien NLP, the average number of clinical concepts and corresponding modifiers identified in a sample of notes from the study using the NLP-NLP protocol was 392. These concepts are coded in various standard terminologies—including ICD-10-CM (International Classification of Diseases, Tenth Revision, Clinical Modification); ICD-9-CM (International Classification of Diseases, Ninth Revision, Clinical Modification); SNOMED-CT (Systematized Nomenclature of Medicine, Clinical Terms); RxNorm; LOINC (Logical Observation Identifiers Names and Codes); and CPT (Current Procedural Terminology)—depending on the type of concept identified. Modifiers are structured data elements that provide additional properties related to a clinical concept. Examples of modifiers include body location, status, and dose (as shown on the right side of Figure 2).

It should be noted that this was a formative study designed to investigate the comparative effects of data capture methods enabled by the NLP system. The focus of the analysis was on characterizing interactive behavior and system usability rather than the NLP method. Future studies will investigate the efficacy of the NLP processes used by the system.

The objectives of this study were to (1) measure the effects, relative to using Standard Entry only, of using 3 NLP-based documentation protocols on EHR documentation time and quality and (2) measure the effects of an NLP Entry–based protocol and a Standard Entry–based protocol on the usability of the documentation process.

## Methods

### Study Overview

The study evaluated an NLP-enabled solution for documentation. Specifically, we focused on three problem areas related to EHR data capture: (1) efficiency, including time required for data capture; (2) effectiveness, encompassing documentation quality; and (3) physician satisfaction, based on usability. We compared a novel dictation-based protocol using MediSapien NLP for structured data capture (“NLP-NLP”) against a standard, keyboard-and-mouse structured data capture protocol where the study participant was instructed to generate EHR documentation as in normal clinical practice (“Standard-Standard”) as well as 2 novel hybrid protocols (“NLP-Standard” and “Standard-NLP”) to determine which protocols provided better results in terms of data capture time, documentation quality, and physician satisfaction. The hybrid protocols were included because we anticipated that mixed forms and modalities of interaction may serve as realistic alternatives to a one-dimensional NLP approach or standard data entry. For example, certain parts of clinical notes may be better served by one modality of entry or the other; a note’s assessment and plan sections are often more given to free text and may therefore be suited to a dictation-based modality, whereas a note’s history and physical examination sections are less so and therefore may be better suited to a different modality. A hybrid approach may offer greater flexibility and can be adapted to the preferences of individual users. The study presented here is formative work that focused more directly on the nature of user interaction and the user experience rather than the efficacy or precision of the NLP system or the system for insertion of data in the EHR. These will be addressed in the next phase of research.

### Study Design

This study contrasted 4 conditions involving combinations of NLP Entry and Standard Entry (referred to in this paper as documentation protocols) on the following measures: documentation time, documentation quality, and usability of the documentation process.

The Standard Entry method (ie, how physicians typically use an EHR to document) entailed using a keyboard and mouse for typing text and negotiating the graphical user interface (eg, drop-down menus, check-boxes) to record information in structured fields.

In the NLP Entry method, the participants dictated the content of the documents. They did not enter any documentation using the keyboard or mouse. Their dictation was transcribed, and the transcription was inputted into the MediSapien NLP application. That application outputted a document containing structured data (an example of which is shown in Figure 2) generated from the transcription. Finally, following precise instructions, study assistants entered the transcribed text and part of the generated structured data into a Microsoft Word document to produce a final note.

**Table 1:** Documentation methods used for each documentation protocol.

Documentation protocols	Documentation method for history and physical examination	Documentation method for assessment and plan
NLP^a^-NLP	NLP Entry	NLP Entry
NLP-Standard	NLP Entry	Standard Entry
Standard-NLP	Standard Entry	NLP Entry
Standard-Standard (control)	Standard Entry	Standard Entry

^a^NLP: natural language processing.

In the study, each physician was asked to document 4 notes using 4 methods including 1 control method (Standard-Standard protocol) and 3 experimental protocols consisting of combinations of NLP Entry and Standard Entry for documenting different parts of the note, as presented in Table 1. The Standard-NLP protocol involved using Standard Entry to generate the history and physical examination sections and NLP Entry to generate the assessment and plan sections. The NLP-Standard protocol involved using NLP Entry for the history and physical examination sections and Standard Entry for the assessment and plan sections. The NLP-NLP protocol involved using NLP Entry for the entire note. The order in which the protocols were used was randomized for each participant.

### Participants

Physician participants were recruited through referrals. Two of the coauthors (BS and PS) referred us to several physicians who in turn made additional referrals. The inclusion criteria for the participants were as follows: (1) must be a neurologist, cardiologist, or nephrologist, the 3 specialties included in the study; (2) must be a senior resident, fellow, or attending; and (3) must be a current user of the Columbia University Medical Center’s (CUMC) Crown Allscripts EHR (Chicago, IL). The participants were each compensated US $500 for their efforts.

### Setting

This study was conducted at CUMC. The test protocol was administered with physician participants at their offices. Fictitious patients were created for the study, and the participants documented their cases in a test environment of the Crown Allscripts EHR, which was the same EHR in which the participants documented during normal clinical practice. Participants were all experienced users of the system. The Crown Allscripts EHR had been in use in excess of 5 years at CUMC as of the time of the study. This test environment contained the same custom templates that participants used during normal clinical practice. As a result, the Standard Entry method simulated documentation during normal clinical practice as closely as reasonably possible.

### Test Scripts

The test scripts were based on anonymized transcription documents that were modified by 4 expert clinicians (2 fellows and 2 attending physicians). These clinicians were not participants in the study. The test scripts consisted of history and physical examination sections but excluded assessment and plan sections. After reviewing test scripts that described cases of the fictitious patients, the participants generated 4 multisectional consultation or admission notes using 4 documentation protocols (Table 1).

### Procedure

**Figure 3:**
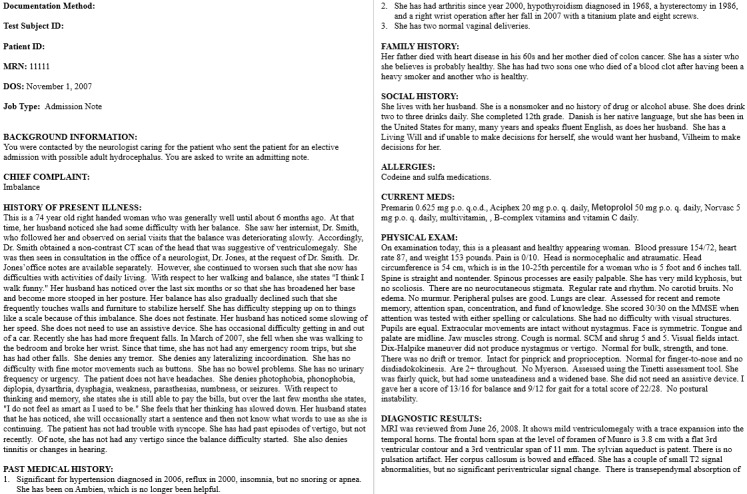
Example of a neurology test script.

First, each participant read the instructions for generating consultation or admission notes based on the 4 provided test scripts, an example of which is shown in Figure 3. The instructions indicated that the participant must generate documentation without copying verbatim any part of the test script and that the assessment and plan sections of these notes would be generated based on the participant’s medical judgment.

**Figure 4:**
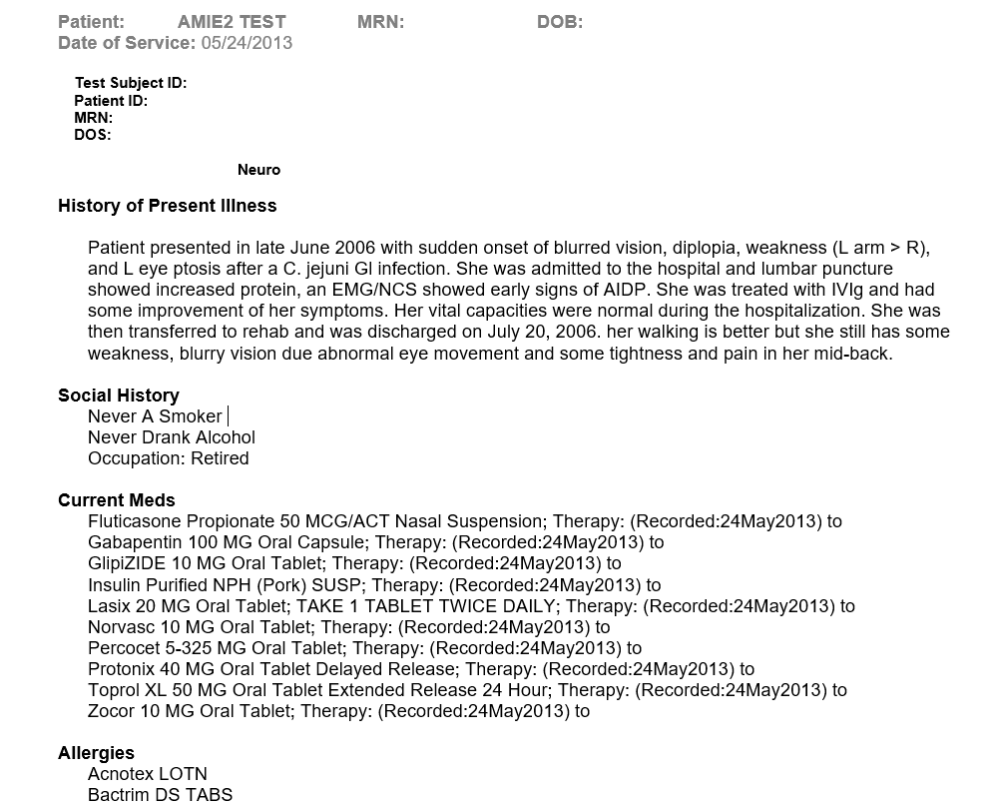
Example of part of the history and physical examination section of a neurology consultation note generated using the Standard-NLP protocol, illustrating the part of the note that was generated by Standard Entry. NLP: natural language processing.

**Figure 5:**
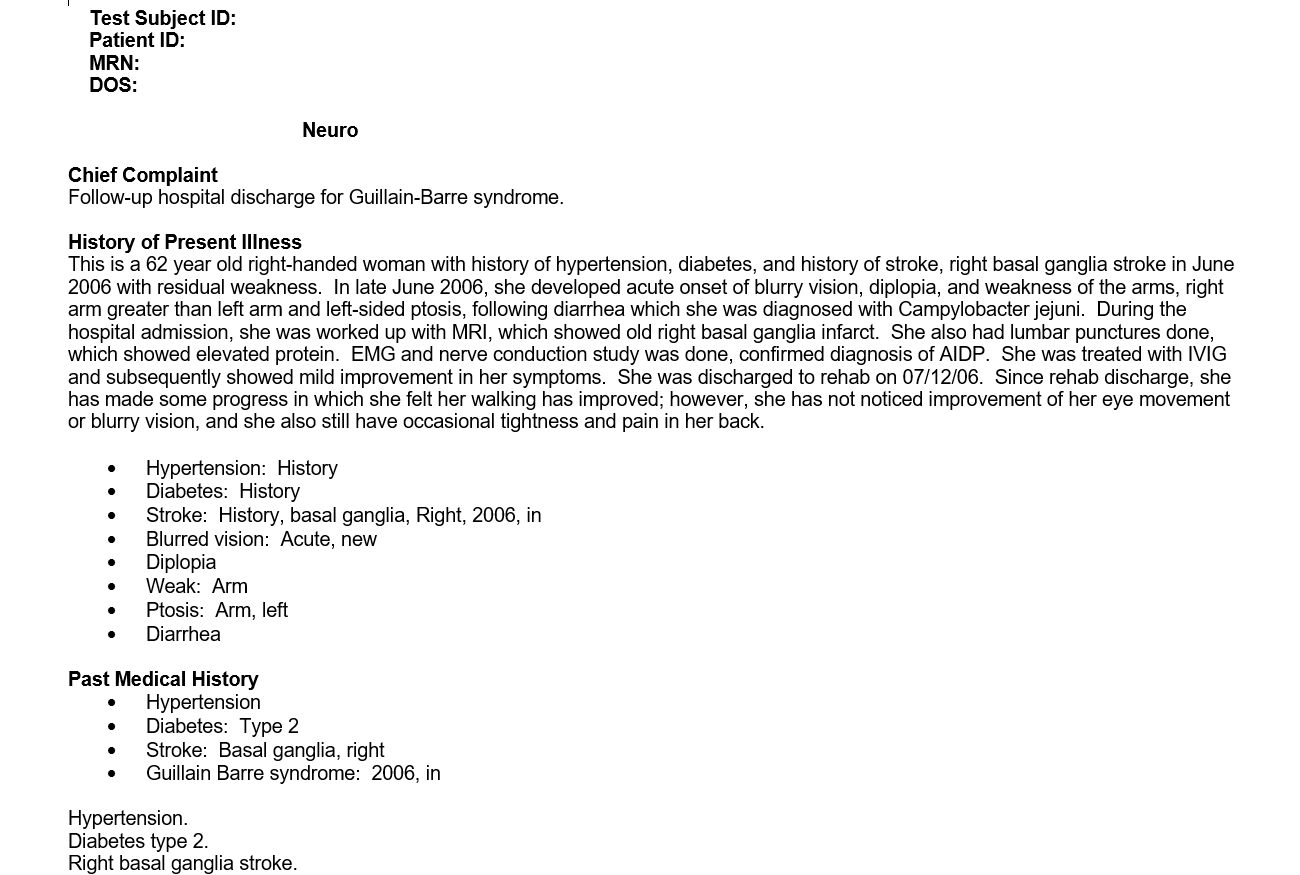
Example of part of the history and physical examination section of a neurology consultation note generated using the NLP-Standard protocol, illustrating the part of the note that was generated by NLP Entry. NLP: natural language processing.

**Figure 6:**
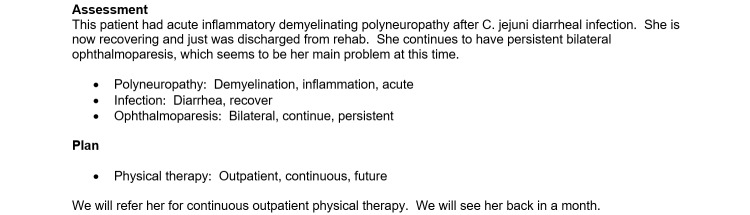
Example of part of the assessment and plan section of a neurology consultation note generated using the Standard-NLP protocol, illustrating the part of the note that was generated by NLP Entry. NLP: natural language processing.

**Figure 7:**
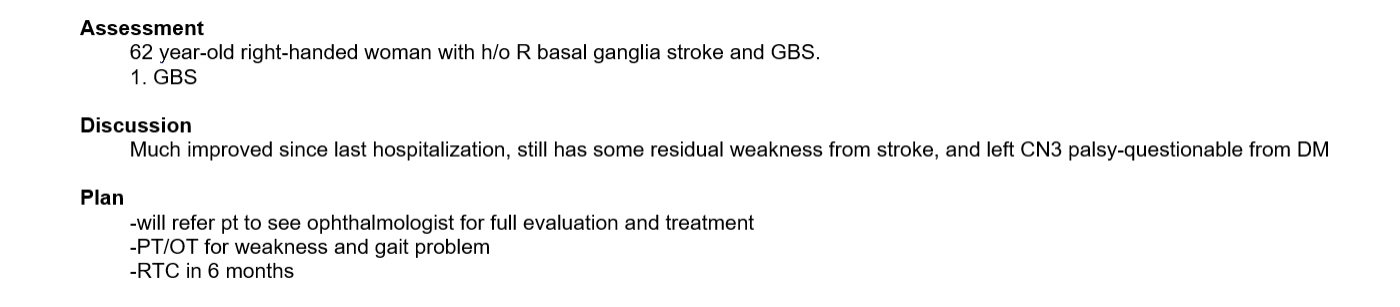
Example of part of the assessment and plan section of a neurology consultation note generated using the NLP-Standard protocol, illustrating the part of the note that was generated by Standard Entry. NLP: natural language processing.

Second, the participant was asked to review the test scripts and to generate 4 notes from 4 test scripts, 4 examples of which are shown in Figures 4-7.

Third, for documentation in which NLP Entry was used, the participant’s dictation was transcribed; the transcription was processed by MediSapien NLP; and the transcription, structured data generated, and any documentation generated for the note by Standard Entry (if applicable) were combined to create the final note. A simulated interface and simulated note were used for NLP Entry: following a protocol, study assistants copied the generated unstructured and structured data into a Microsoft Word document to generate the final note. For Standard Entry, an actual EHR interface was used.

Finally, after reviewing their final notes, the participants completed 2 System Usability Scale (SUS) surveys [37] to evaluate the usability of the NLP-NLP protocol and the Standard-Standard protocol. Given the limited availability of clinicians’ time, we determined this would be the most important contrast to include in the study. The SUS is a widely used and reliable tool. It consists of 10 Likert items measured on a 5-point scale (ranging from “completely agree” to “completely disagree”) [38]. Half of the items are framed as positive questions (eg, “easy to use”) and half are negative (eg, “unnecessarily complex”). The scores were tabulated accordingly. The surveys were made available in SurveyMonkey, a Web-based survey application. The SUS was slightly modified for language and context. The questions with tabulated responses are presented in Table 2.

**Table 2:** Summary of usability scores (mean, SD) and paired *t* test comparisons between use of the Standard-Standard and the NLP-NLP protocols (n=23 cases); scores have been normalized such that higher scores indicate greater usability.

Usability question	Standard-Standard, mean (SD)	NLP^a^-NLP, mean (SD)	*P* value
I think that I would like to use this method frequently for admitting notes.	2.9 (0.9)	3.3 (0.8)	.21
I found this method unnecessarily complex.	2.5 (1.4)	3.8 (0.8)	.003
I thought this method was easy to use.	2.8 (1)	4.2 (0.6)	<.001
I think that I would need assistance to be able to use this method.	3.3 (1.1)	3.6 (0.9)	.24
I found the various functions in the processes of the method were well integrated.	2.6 (0.9)	3.2 (1)	.05
I would imagine that most people would learn to use this method very quickly.	3.0 (0.9)	3.8 (0.7)	.01
I found this method very cumbersome/awkward to use.	2.6 (1.1)	3.7 (0.9)	.004
I felt very confident using this method.	3.6 (0.8)	3.4 (0.8)	.43
I would need to learn a lot of things before I could get going with this method.	3.6 (1)	3.8 (0.8)	.40
I feel the method would fit well in my existing workflow.	2.8 (0.9)	3.4 (0.9)	.08

^a^NLP: natural language processing.

### Measures of Analysis

#### Time, Documentation Quality, and Usability

The time required for documentation was measured using a stopwatch.

**Figure 8:**
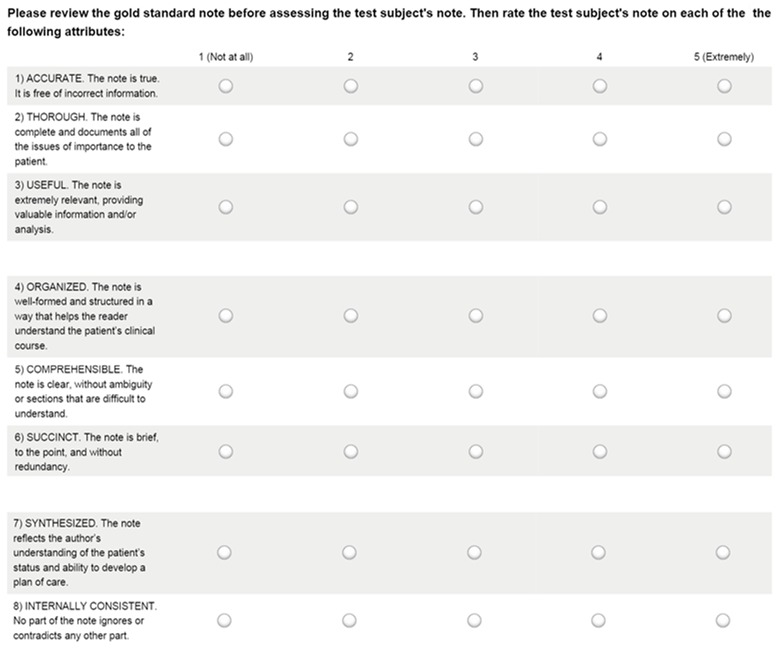
Physician Documentation Quality Instrument (PDQI-9) tool.

The 4 expert clinicians (2 fellows and 2 attending physicians) were not participants and were blind to the protocols used to generate the documentation they evaluated. They were provided with gold standard versions of the test documentation they were asked to evaluate and told that the gold standard versions represented “high quality notes.” They were then instructed to measure documentation quality by comparing participants’ final test documentation against the gold standard versions of that documentation using the PDQI-9 tool [21], shown in Figure 8. The expert clinicians were given minimal background on the purpose for the study. They were independent and highly trained in their specialties (and they only graded documents within their own domains). In addition, they were provided with clear instructions and had a sound understanding of the PDQI-9 tool, which is known to be a reliable instrument [21]. Unfortunately, it was not practical to test interrater reliability. We used only 8 of the 9 PDQI-9 prompts (items) because one of the prompts required a judgment of whether the documentation was up-to-date and this was not meaningful in this particular context.

The gold standard versions of the documentation were generated by the expert clinicians in Microsoft Word. They produced these documents from clinical notes and modified them so that they were consistent with the clinical profile of the patient (ie, the patient’s assessment and treatment were consistent and derivable from history and physical examination findings). The expert clinicians were instructed to ensure that all elements of the documents were internally consistent and that they truly reflected a gold standard. The expert clinicians were compensated at a rate of US $125 per hour for their efforts. We did not have access to the interim work product of the expert clinicians. We were only provided with the expert clinicians’ grades.

The usability of the documentation processes was assessed using a modified version of the SUS [38]. Each participant completed 1 SUS questionnaire for each of the NLP-NLP and Standard-Standard protocols.

#### Statistical Analysis

Data were analyzed using Intercooled Stata version 9.2 (StataCorp LP). Demographics were tabulated in regard to participants’ years of EHR experience, years of experience with dictation, the number of cases per subject area, the frequency of use of each of the 4 protocols, dictation time, usability scores, and quality scores. The Shapiro-Wilk W test was used to determine whether continuous variables were normally distributed. Results are presented as mean (SD) or analysis of variance (ANOVA) results, median (interquartile range), or percentage; respectively, comparisons were made using *t* test, Wilcoxon rank sum analysis, or chi-square analysis.

Pearson correlation was performed on continuous variables. The association of years of EHR experience, years of experience with dictation, the 4 protocols (Standard-Standard, Standard-NLP, NLP-Standard, and NLP-NLP), and the 3 subject areas (cardiology, nephrology, and neurology) with the note dictation time was assessed using ANOVA.

Statistical significance was defined as alpha=.05 and Bonferroni correction was used where applicable for multiple comparisons.

### Human Subjects Protection

The study was approved by the Institutional Review Board at Columbia University (#AAAK2458). All participants gave consent before their participation and were fully briefed on the true objectives of the study. The study protocol adhered to strict standards of confidentiality and privacy.

## Results

A total of 31 unique individuals documented 3.8 (SD 0.7) notes on average. Of these, 28 participants completed all 4 protocols, 2 participants completed 2 protocols, and 1 participant completed 1 protocol. The participants who did not complete all 4 protocols were called away from the study and therefore could not finish the task. These individuals had an average EHR experience of 6.6 (SD 3.4) years (data were available for 30 individuals) and an average dictation experience of 2.8 (SD 5.6) years (data were available for 29 individuals). There was a significant association between years of EHR experience and years of dictation experience (*r*=.47, *P*=.01).

A total of 118 notes were documented across the 3 subject areas of cardiology (22/118, 18.6%), nephrology (21/118, 17.8%), and neurology (75/118, 63.6%). The Standard-Standard, Standard-NLP, NLP-Standard, and NLP-NLP protocols were used in 28/118 (23.7%), 28/118 (23.7%), 30/118 (25.4%), and 32/118 (27.1%) documented notes, respectively. The frequency of use of the 4 protocols was balanced across the 3 subject areas (see Table 3).

**Table 3:** Frequency of use of the 4 protocols by subject area for each documented note.

Protocol	Documented cardiology notes, n (%)	Documented nephrology notes, n (%)	Documented neurology notes, n (%)	Total number of documented notes, n (%)
Standard-Standard	5 (23)	5 (24)	18 (24)	28 (23.7)
Standard-NLP^a^	5 (23)	4 (19)	19 (25)	28 (23.7)
NLP-Standard	6 (27)	5 (24)	19 (25)	30 (25.4)
NLP-NLP	6 (27)	7 (33)	19 (25)	32 (27.1)
Total	22	21	75	118

^a^NLP: natural language processing.

**Table 4:** Median documentation time in minutes, with interquartile ranges, by protocol and subject area.

Protocol	Median (IQR^a^) time to document cardiology note (minutes)	Median (IQR) time to document nephrology note (minutes)	Median (IQR) time to document neurology note (minutes)
Standard-Standard	16.9 (16.5-19.7)	20.7 (18.6-23.2)	21.2 (17.6-29.9)
Standard-NLP^b^	13.8 (13.0-17.2)	21.3 (14.5-29.8)	18.7 (16.0-22.9)
NLP-Standard	7.5 (7.1-9.1)	12.1 (10.7-12.2)	11.0 (8.5-14.6)
NLP-NLP	5.2 (4.7-8.0)	7.3 (6.6-9.1)	8.5 (6.4-11.4)

^a^IQR: interquartile range.

^b^NLP: natural language processing.

**Table 5:** Interprotocol comparisons (Wilcoxon rank sum analysis).

Interprotocol comparisons	Statistical analysis of time difference (*P* value^a^)		
	Cardiology notes	Nephrology notes	Neurology notes
Standard-Standard vs Standard-NLP^b^	.60	.81	.20
Standard-Standard vs NLP-Standard	.01	.03	<.001
Standard-Standard vs NLP-NLP	.006	.005	<.001
Standard-NLP vs NLP-Standard	.006	.05	.001
Standard-NLP vs NLP-NLP	.006	.008	<.001
NLP-Standard vs NLP-NLP	.11	.02	.02

^a^Statistical significance level: alpha=.0083 after Bonferroni correction.

^b^NLP: natural language processing.

**Table 6:** Document quality for each protocol (median values are presented).

Protocols and statistical comparisons		Document quality metrics^a^								
		A	T	U	O	C	S	Sy	I	Sum
**Protocol, median score**										
	Standard-Standard (n=24)	3.5	3	4	4	4	4	4	4	29
	Standard-NLP^b^ (n=24)	4	4	4	3.5	4	2.5	4	4	29.5
	NLP-Standard (n=27)	4	3	3	3	3	3	4	4	26
	NLP-NLP (n=30)	4	4	3	3	3	2	3	4	24.5
**Interprotocol comparisons,** ***P* value^c^**										
	Standard-Standard vs Standard-NLP		.04		.03		<.001			
	Standard-Standard vs NLP-Standard					.04	.006			
	Standard-Standard vs NLP-NLP				.002	.02	<.001	.03		
	Standard-NLP vs NLP-Standard						.005			
	Standard-NLP vs NLP-NLP							.02		
	NLP-Standard vs NLP-NLP				.03		.001			

^a^The 8 document quality metrics are as follows: Accurate, Thorough, Useful, Organized, Comprehensible, Succinct, Synthesized, and Internally Consistent.

^b^NLP: natural language processing.

^c^Statistical significance level: alpha=.0083 after Bonferroni correction.

The documentation times were not normally distributed (*z*=4.6); thus, comparison of documentation times was performed using Wilcoxon rank sum analysis of medians. Table 4 summarizes the median time and interquartile range for the documentation of each note by protocol and subject area. Table 5 presents statistical analysis of interprotocol times. In each subject area, the NLP-NLP protocol required significantly less documentation time compared with either the Standard-Standard or Standard-NLP protocol. Compared with the Standard-Standard protocol, the NLP-Standard protocol required significantly less documentation time for the neurology subject area. Compared with the Standard-NLP protocol, the NLP-Standard protocol required significantly less documentation time for the cardiology and neurology subject areas. 

On the basis of the ANOVA of documentation time, the model was statistically significant (adjusted *R*^2^=.54, *P*<.001). This indicates that, taken together, the input variables used in the ANOVA model (EHR experience; years of experience with dictation; the 4 protocols, Standard-Standard, Standard-NLP, NLP-Standard, and NLP-NLP; and the 3 subject areas, cardiology, nephrology, and neurology) accounted for 54% of the variance in documentation time, the outcome variable. The factors significantly associated with documentation time included the protocol method (*P*<.001), subject area (*P*=.009), and the number of years of EHR experience (*P*=.047) but not the number of years of dictation experience (*P*=.77).

Document quality was assessed using 8 observed PDQI-9 metrics (Figure 8). Median values of the document quality metrics are presented for each protocol, in Table 6. Statistical comparisons across the protocols are also presented in Table 6. The significant differences among the protocols occurred within the “Organized” metric (Standard-Standard vs NLP-NLP, 4 vs 3, *P*=.002) and the “Succinct” metric (Standard-Standard vs Standard-NLP, 4 vs 2.5, *P*<.001; Standard-Standard vs NLP-Standard, 4 vs 3, *P*=.006; Standard-Standard vs NLP-NLP, 4 vs 2, *P*<.001; Standard-NLP vs NLP-Standard, 2.5 vs 3, *P*=.005; and NLP-Standard vs NLP-NLP, 3 vs 2, *P*=.001).

The usability data were analyzed using a paired *t* test in a subset of 23 cases (n=5 cardiology, n=5 nephrology, and n=13 neurology) in which the same participant documented a case with both Standard-Standard and NLP-NLP protocols. The average duration of EHR experience for these 23 individuals was 6.3 (SD 2.8) years. The total score of the 10-component SUS measure was significantly higher when the participants used the NLP-NLP protocol compared with when they used the Standard-Standard protocol (mean 36.7, SD 5.4, compared with mean 30.3, SD 7.7; *P*=.007). Table 2 summarizes the usability scores and paired comparisons between the 2 protocols. Responses to 4 of the 10 usability questions (complexity, ease of use, learning the method very quickly, and cumbersomeness or awkwardness of use) significantly favored the NLP-NLP protocol over the Standard-Standard protocol.

## Discussion

### Findings

This formative study sought to assess the feasibility of using an EHR documentation method based on dictation and NLP by evaluating the effect of the method on documentation time, documentation quality, and usability. We found that a pure protocol of NLP Entry as well as hybrid protocols (involving both NLP Entry and Standard Entry) showed promise for EHR documentation, relative to Standard Entry alone (Standard-Standard Entry). It is our opinion that different parts of the note should be documented differently, but reaching a conclusion on the optimal method of documentation for each part of the note will require further study.

The finding that NLP-NLP Entry and NLP-Standard Entry required significantly less time than Standard-Standard Entry can be explained by the faster speed of dictation relative to that of entering data using the keyboard and mouse, rather than by the involvement of NLP.

No statistically significant difference was found between the overall documentation quality (measured using the PDQI-9 tool) of Standard-Standard Entry and that of any of the other 3 documentation protocols. The succinctness of Standard-Standard Entry documentation was found to be significantly greater than that of the other 3 protocols. This suggests that the note was judged to be more to the point and with less redundancy. In addition, documentation from Standard-Standard Entry was found to be more organized than that from NLP-NLP Entry, indicating that it was structured in a way that the reader could better understand the patient’s clinical course. When the participant used the Standard-Standard protocol, they used Standard Entry for history and physical examination sections as well as assessment and plan sections. When they used the Standard-NLP protocol, they used Standard Entry for history and physical examination sections and NLP Entry for assessment and plan sections. In the former (Standard-Standard), the participants tended to type shorter paragraphs for the assessment and plan sections. In the latter (Standard-NLP), they dictated the assessment and plan resulting in a larger volume of text. This difference warrants future scrutiny. On the basis of the results of the modified SUS, the participants’ usability ratings for NLP-NLP Entry were significantly higher than for Standard-Standard Entry. These findings suggest that, pending further study, EHR documentation methods using a combination of dictation and NLP show potential for reducing documentation time and increasing usability while maintaining documentation quality, relative to EHR documentation via standard keyboard-and-mouse entry.

Documentation methods using dictation and NLP have the potential to reduce some of the most egregious “pain points” for EHR data entry. These methods can facilitate capture and insertion of both structured data and transcribed text into the appropriate EHR sections, affording the user of the note the option of using one or both types of information. The structured data are ideal for interoperability and coding and may prove to be useful for analytics.

Opinion is divided regarding the relative advantages of narrative fields and structured fields in clinical documentation and in which contexts each excels or is preferable [39,40]. The flexibility to allow providers to enter text in narrative fields clashes with the desire to produce structured data to facilitate reuse of this information in EHRs [22]. It is this quest for achieving a balance of expressivity, richness, and completeness of detail in the patient health story, with reusable, and thus actionable, structured data in the patient EHR that motivates this field of inquiry. Improving the quality of structured notes generated within an EHR from the structured output of NLP Entry and transcribed text is an area requiring further study and development work.

### Future Research

In future research, for the purpose of achieving documentation quality using dictation and NLP that, in all respects, is comparable to or better than documentation quality resulting from Standard Entry, certain changes to the NLP Entry process will be evaluated. We will assess the effects of requiring participants to use dictation under the constraints of a structured template on improving the organization, comprehensibility, succinctness, and synthesis of notes produced from NLP Entry. The templates would reflect the structure of the participant’s EHR. In addition, we will aim to improve the procedures by which NLP output data are translated into and transferred to the clinical note. We also plan to more systematically scrutinize data capture differences pertaining to documenting in different sections of the EHR note. This will enable us to fine-tune hybrid methods of data entry.

In a subsequent study, we will measure the time required for, and documentation quality and usability of, NLP Entry in live clinical use. This will require developing automated interfaces for sending the participants’ dictation to MediSapien NLP and for sending structured data and free text from MediSapien NLP into the EHR, during which process the participant will be able to modify the documentation. This process will be facilitated by the emergence and widespread adoption of interoperability standards and messages that can carry rich structured data.

### Limitations

This study has several limitations. One limitation is that the simulated interface used in this controlled experiment is somewhat lacking in ecological validity. In a real-world live setting, the structured data and the transcribed text data would both be inserted into the EHR via an automated interface. In addition, the physician would be able to review or modify the documentation before it was finalized. For the purposes of this formative study, this process was simplified. Therefore, an interface to automatically insert the structured data and text into the EHR or allow the physician to review the documentation before finalization was not used for this study. Instead, the insertion process was simulated by manually generating a note in Microsoft Word resembling one that might have been generated by the automated insertion process. Time required for generating the note was not included in the study’s time measurements. To ensure that the manually generated note could have been produced by an automated process, it was produced following strict predetermined rules and without any reliance on human discretion.

Second, physicians generated documentation for the study based on test scripts about fictitious patient encounters. Test scripts included history and physical examination sections and were formatted as transcription notes. The assessment and treatment plan sections were excluded from the test scripts. Participants were instructed not to dictate or type verbatim what was written in the test scripts, but to understand what was written and document it in their own way. In addition to being instructed to generate history and physical examination sections, they were instructed to generate their own assessment and treatment plan sections, because those sections were excluded from the test script.

The sample size for cardiology and nephrology was rather small owing to recruiting challenges. This affected the power for determining differences for related contrasts. Clearly, a larger sample size would have enabled us to detect more subtle group differences.

A limitation of this method of generating test documentation was that because it presented medical information in a free-text format, it may have favored documentation methods requiring the physician to generate free text. NLP Entry requires documentation via dictation exclusively, and Standard Entry entails only some documentation via typing, with the rest entered by pointing and clicking using a mouse. Consequently, the results for time required to complete documentation may be biased toward free text and therefore toward NLP Entry. Nevertheless, we perceive a value in measuring the temporal differences and think that such differences may be consequential in real-world use of this system.

### Conclusions

Current standard methods of EHR documentation have been shown to be extremely time consuming and are judged to have suboptimal usability. In this formative study, the feasibility of an approach to EHR data capture involving applying NLP to transcribed dictation was demonstrated. This approach was shown to have the potential to reduce the time required for documentation and improve usability while maintaining documentation quality in several respects. Future research will evaluate the NLP-based EHR data capture approach in a live clinical setting where generated structured data and transcribed text for real patients are inserted into the EHR via an automated interface.

The past decade has witnessed a dramatic increase in the adoption of EHRs as central instruments in medical practice. However, these systems have not yet proven to be reliable tools for facilitating clinical workflow or enhancing patient care. Recent advances in usability have led to the development of frameworks, new methods, and robust assessment tools that can be used to more precisely delineate the source of the problems associated with an interface [41,42]. In addition, novel approaches to design have provided new EHR approaches that better support flexibility and expressivity [43]. We anticipate that NLP-enabled data entry tools will form an important part of the solution space and will serve to enhance the user’s experience.

There is ample evidence that clinicians spend many hours documenting patient records and sometimes at the expense of time that could be devoted to patient care. Dictation is a familiar method of data entry to most clinicians. The proposed solution leverages that familiarity and has the potential to produce a quality document or patient note in less time along with highly structured machine-readable codes.

## Abbreviations

ANOVA: analysis of variance
CBD: computer-based documentation
CUMC: Columbia University Medical Center
EHR: electronic health record
HITECH: Health Information Technology for Economic and Clinical Health
MedLEE: Medical Language Extraction and Encoding System
NLP: natural language processing
PDQI: Physician Documentation Quality Instrument
SUS: System Usability Scale
